# Developing a multidimensional pedagogical framework for safety education in early childhood: mapping systematic review insights to *Sheriff Labrador*

**DOI:** 10.3389/fpubh.2026.1765782

**Published:** 2026-03-13

**Authors:** Olusiji Adebola Lasekan, Margot Teresa Godoy Pena, Blessy Sarah Mathew

**Affiliations:** 1 Departamento de Educación e Innovación, Universidad Católica de Temuco, Temuco, Chile; 2 Languages Coordination, DITFO, Universidad de La Frontera, Temuco, Chile; 3 Department of Economics, Lovely Professional University, Phagwara, Punjab, India

**Keywords:** content analysis, early-childhood safety education, instructional media, pedagogical framework, *Sheriff Labrador*

## Abstract

Early-childhood safety education is essential for reducing children’s vulnerability to everyday hazards, yet existing approaches remain fragmented and lack comprehensive pedagogical structure. This study integrates evidence from a systematic literature review (SLR) and media-based content analysis to develop a multidimensional framework for safety instruction. First, the SLR identified five overarching dimensions of early-childhood safety education—content, pedagogical, developmental, contextual, and implementation—highlighting the complexity of safety learning and the need for coordinated instructional strategies. Second, a thematic content analysis of 35 episodes of the animated series *Sheriff Labrador* examined how safety content is represented across seven domains: physical environment safety, traffic and pedestrian safety, water safety, personal safety and protection, emergency preparedness, health and hygiene, and rural and agricultural safety. The results showed broad alignment between media representations and research-derived safety domains, demonstrating the program’s potential as an instructional resource. Synthesizing findings from both phases, the study developed the Multidimensional Pedagogical Safety Integration Model (MPSIM), a theory-informed framework grounded in Social Learning Theory, Ecological Systems Theory, Constructivist developmental principles, Behavioral Skills Training, and Experiential Learning Theory. The model provides a structured process for determining what to teach, how to teach it, for whom, under what conditions, and how to sustain learning. Implications for educators, curriculum developers, and policy frameworks are discussed.

## Introduction

Early childhood safety education is a critical component of children’s development, laying the foundation for lifelong safety awareness and behavior. As young children are among the most vulnerable members of society, equipping them with safety knowledge and skills helps them navigate diverse environments and reduces the risk of accidents and injuries (1, 2). Such education promotes the understanding of potential hazards and fosters safe habits early in life, including traffic safety awareness, which is essential given children’s frequent exposure to road environments (3). In addition, disaster-preparedness education empowers children to respond effectively to emergencies, ensuring readiness for real-life events (4). Effective safety instruction relies on interactive, engaging approaches—such as games, visual media, and role-playing—to enhance retention and meaningful learning (3, 5), while the integration of technology further enriches context-based understanding (6). Community and family involvement reinforces school-based lessons, helping cultivate a broader culture of safety (3). However, successful implementation depends on well-trained educators and sensitivity to cultural and socioeconomic factors, which can influence how safety education is delivered and received (7). Overall, early-childhood safety education contributes to safer learning environments and supports children’s holistic well-being.

The lack of structured pedagogical frameworks in early childhood safety education poses significant challenges to educators’ ability to effectively teach essential safety skills to young children. This issue is particularly pressing given the diverse range of safety topics—physical, digital, social, and road-related—that must be addressed in developmentally appropriate ways. Although emerging initiatives demonstrate the value of systematic approaches, including the Safety Matters Training Program in Harbin, China, which significantly improved teachers’ competencies (7), and Australia’s National Practices for Early Childhood Road Safety Education aligned with the Early Years Learning Framework (2, 8), existing frameworks remain fragmented and context-specific. Reviews also highlight the need for tailored digital safety frameworks for pre-schoolers, underscoring the importance of age-appropriate guidance (9). Compounding this gap are challenges such as the breadth of safety content requiring varied instructional strategies (3, 10), cultural and socioeconomic factors influencing implementation (11), and limited systematic evaluation of teaching methodologies (10). Proposed solutions include the use of interactive and engaging methods—such as games and visual media—to support safety learning (3), greater parental and community involvement to reinforce practices beyond the classroom (12), and simulation-based training to better prepare caregivers and educators (12). Together, these findings highlight a pressing need for comprehensive, evidence-based pedagogical frameworks capable of supporting effective safety education in early childhood contexts.

*Sheriff Labrador* has emerged as a valuable instructional media resource for promoting early-childhood safety education because it embeds safety lessons within developmentally appropriate narratives that young children can easily understand and internalize. As a popular animated children’s series, it uses colorful visuals, simple dialogue, and relatable characters to sustain attention while conveying essential safety principles. In each episode, *Sheriff Labrador* models safe behavior by identifying hazards, explaining risks, and guiding children through appropriate responses—often using clear verbal instruction, visual demonstrations, and step-by-step problem solving. The sheriff typically introduces a safety issue, investigates the cause of the danger, and then delivers corrective guidance that reinforces key safety rules, such as avoiding unsafe objects, interacting safely with strangers, or behaving appropriately near traffic or water. These lessons are integrated into engaging storylines in which unsafe behavior leads to identifiable consequences, while safe behavior results in protection and positive outcomes. By combining storytelling, observation, explicit instruction, and repeated messaging, this animated series offers an accessible and appealing medium for young learners, making it a promising supplement to classroom-based safety education.

*Sheriff Labrador* was selected for analysis based on three primary considerations. First, the series represents a structured form of “edutainment,” a genre that intentionally blends entertainment narratives with explicit instructional objectives (13). Unlike purely recreational animation, *Sheriff Labrador* systematically centers each episode around a clearly defined safety problem, followed by modeled corrective behavior and explicit verbal instruction. Second, preliminary screening indicated consistent and repeated coverage of diverse safety domains—including fire safety, hygiene, road safety, and environmental hazards—making the series analytically suitable for multidimensional mapping against empirically derived safety frameworks. Third, the program’s pedagogical structure—problem identification, modeling, explanation, and resolution—mirrors established instructional sequences such as Behavioral Skills Training and Social Learning processes (14, 15), thereby providing a theoretically relevant case for examining how media content can be systematically integrated with instructional design. For these reasons, the series was purposively selected as an illustrative model of media-based safety education rather than as a randomly chosen example.

Despite growing recognition of the importance of early-childhood safety education, there remains a critical research gap in the integration of children’s media content into pedagogical practice. While animated programs such as *Sheriff Labrador* explicitly convey safety lessons through modeling, narration, and problem-solving, these media resources are rarely examined systematically or linked to structured instructional frameworks for classroom use. Existing research focuses primarily on identifying safety topics or evaluating educational interventions, yet little attention has been given to how media-based safety messaging can be aligned with evidence-based pedagogical, contextual, developmental, and implementation considerations in early-childhood settings. As a result, media content that already embeds safety knowledge remains underutilized in formal education, limiting its potential impact on children’s learning. Addressing this gap requires an approach that first clarifies the multidimensional structure of safety education, then analyzes how *Sheriff Labrador* expresses content dimension, and finally organizes these insights into a process-based pedagogical model to guide instructional application. This study therefore aims to: (1) identify the multidimensional domains of early-childhood safety education through systematic review; (2) examine how content dimension are represented in *Sheriff Labrador* through thematic content analysis; and (3) construct a pedagogical framework integrating content, pedagogical, contextual, developmental, and implementation dimensions.

The present study is conceptually grounded in several complementary learning theories that explain how young children acquire and apply safety behaviors. Social Learning Theory (14) highlights the importance of modeling and observational learning in behavior acquisition, particularly relevant in animated media contexts. Ecological Systems Theory (16) situates children’s safety learning within nested environmental systems, including home, school, and community contexts. Constructivist developmental perspectives (17, 18) emphasize the need for age-appropriate scaffolding and concrete learning experiences. Behavioral Skills Training (15) and Experiential Learning Theory (19) further underscore the role of structured instruction, rehearsal, and reflection in skill mastery. These theoretical perspectives provide the conceptual lens through which safety education content and media representations are analyzed and synthesized.

## Literature review

Early-childhood safety education is a critical component of child development, designed to equip young learners with the knowledge and skills needed to navigate hazards safely. The rationale for this emphasis stems from children’s heightened vulnerability to unintentional injuries—resulting from developing motor skills, curiosity, and limited experience—making injuries a leading cause of childhood morbidity and mortality, particularly from falls, burns, and drowning (20, 21). Research indicates that preventive safety education can meaningfully reduce these risks by improving children’s safety knowledge, skills, and attitudes, ultimately decreasing injury incidence (22). Foundational work in this area highlights diverse hazards in domestic and community settings—including traffic risks, household accidents, stranger danger, and abuse—which necessitate developmentally appropriate teaching approaches (10). Recommended instructional strategies include storytelling, role-playing, and interactive activities to enhance comprehension and engagement (23), supported by curriculum integration to ensure continuity of learning (20). The literature further underscores the shared responsibility of multiple stakeholders: parents reinforce safety practices in the home, educators implement structured programs tailored to learner needs, and community and health professionals contribute resources and expertise to strengthen program delivery (24). Collectively, these perspectives highlight the multidimensional nature of early-childhood safety education and the importance of coordinated efforts to promote safer environments for young children.

Animation plays a multifaceted role in children’s learning, serving educational, safety, and creative functions that make it a powerful instructional medium. Research shows that animated content—particularly 3D formats—can significantly enhance learning outcomes in subjects such as science by helping children visualize and grasp complex concepts in an engaging manner (25). Animation also fosters creativity, as demonstrated by approaches that transform children’s drawings into animated stories—such as retellings of “Timun Emas”—which nurture imagination while reinforcing educational values (26). In addition, animations have been effectively used to teach programming logic, demonstrating the medium’s ability to support critical thinking development in young learners (27). Beyond academic subjects, animation is widely applied in safety-focused media; web-based interactive 2D and 3D animations help children understand safety rules by aligning instructional content with children’s developmental and psychological characteristics (28). Animated videos have also proven valuable in marginalized communities, improving preschoolers’ body safety awareness and highlighting the benefits of multimodal protective education (29). Moreover, animations support disaster preparedness: fire-safety videos allow children to visualize emergency situations and appropriate responses, contributing to improved readiness (30). Their utility further extends to environmental and hygiene education, where animated content effectively promotes responsibility, cleanliness, and healthy habits (31, 32). Collectively, these studies demonstrate that animation is an engaging, developmentally appropriate medium capable of supporting diverse learning goals, including safety, creativity, and foundational knowledge.

Integrating safety education into a TV show for young children requires thoughtful use of engaging, developmentally appropriate strategies that enhance understanding and retention of key concepts. Research demonstrates that interactive and multimedia approaches—such as videos, songs, and narrative storytelling—effectively support children’s learning, as seen in disaster-learning models that improved preparedness among preschoolers (33). Digital tools, including augmented-reality applications like Kinder, further enable children to identify environmental hazards and build practical safety awareness (34). Narrative-based pedagogies also play an important role: fairy-tale–based situational role-playing has been shown to strengthen children’s safety knowledge and problem-solving abilities (35), while puppetry and storytelling have been used successfully to teach fire safety and drowning prevention by engaging children emotionally and making lessons memorable (36, 37). Visual and reflective strategies—such as animated videos, posters, and guided reflection—have proven effective in reinforcing safety behaviors, particularly in early-childhood fire safety and body-protection education among marginalized groups (29, 30). Broader curriculum-based frameworks, including structured road-safety programs like VicRoads, illustrate how sequenced safety instruction can be adapted into broadcast formats to promote lifelong safe behavior (2). Complementary teacher-training initiatives, such as the Safety Matters Training Program, underscore the importance of structured learning objectives and expert guidance—elements that can be mirrored in TV content to enhance instructional quality (7). Collectively, this literature highlights that engaging narratives, interactive media, and structured pedagogical design can make TV programming an effective vehicle for early-childhood safety education.

Educational television has long been recognized as a powerful medium for cognitive and socio-emotional development in young children. Foundational research on programs such as *Sesame Street* demonstrated that carefully designed media can enhance literacy, numeracy, and prosocial behaviors when instructional goals are embedded within engaging narratives (38, 39). The entertainment–education paradigm further established that media narratives can intentionally promote behavioral change through modeling, emotional engagement, and repeated exposure (40, 41). Within this tradition, edutainment media are designed not merely to inform but to integrate pedagogical objectives with narrative storytelling (13).

In the context of children’s safety programming, media-based interventions have been used to teach fire prevention, drowning awareness, disaster preparedness, and injury prevention skills (30, 36, 37). These studies demonstrate that animated and performative formats can enhance retention of safety procedures by combining visual modeling, repetition, and emotional salience. Research in injury prevention further emphasizes the role of behavior modeling and guided rehearsal in promoting protective behaviors among young children (42). Despite this established lineage, few studies have systematically mapped a contemporary animated safety series onto empirically derived instructional dimensions or integrated such media within a structured pedagogical framework. The present study extends the educational media tradition by situating *Sheriff Labrador* within this lineage and examining how its safety representations can be aligned with a multidimensional, theory-informed instructional model.

The term *safety education* has been used in varied ways across the literature, often reflecting different disciplinary emphases. From an injury-prevention perspective, safety education is defined as structured efforts to reduce children’s exposure to environmental hazards and prevent unintentional injury through risk awareness and behavioral modification (21, 42). A behavioral skills perspective conceptualizes safety education as the explicit teaching of observable protective competencies—such as refusing unsafe requests, performing evacuation procedures, or identifying hazardous objects—often delivered through modeling and rehearsal (10, 15). More recent scholarship adopts a holistic developmental view, framing safety education as a broader process that integrates cognitive understanding, socio-emotional regulation, contextual awareness, and family–community engagement (1, 2). In this study, safety education is conceptualized as a structured, developmentally aligned process through which young children acquire hazard recognition skills, protective behavioral competencies, emotional regulation strategies, and contextual awareness necessary to reduce injury risk and promote well-being.

Similarly, the term *instructional framework* is frequently conflated with curriculum or intervention programs. Whereas a curriculum typically specifies content sequences and learning outcomes, and interventions refer to applied programmatic implementations, an instructional framework provides an organizing structure that integrates content, pedagogy, context, and assessment within a coherent theoretical system (20, 43). Within this study, an instructional framework refers to a theory-informed, multidimensional structure that systematically connects what is taught, how it is taught, for whom, under what contextual conditions, and through what mechanisms learning is sustained.

Finally, the term *multidimensional* is used analytically rather than descriptively. While many safety education studies focus on single domains—such as fire prevention or traffic safety—others emphasize isolated pedagogical techniques or contextual factors. Few integrate these domains into a unified system. Drawing conceptually from ecological systems theory (16) and multi-level injury prevention models (42), the present study defines multidimensional safety education as an integrated model that synthesizes interacting domains of content, pedagogy, development, context, and implementation. This multidimensional conceptualization forms the foundation for the development of the Multidimensional Pedagogical Safety Integration Model (MPSIM).

## Theoretical foundations of early-childhood safety education

Effective early-childhood safety education is not only content-driven but also theoretically grounded in established learning and developmental frameworks that explain how young children acquire, internalize, and generalize safety behaviors. Among these, Social Learning Theory (14) provides a foundational explanation for how children learn safety skills through observation, imitation, and vicarious reinforcement. Young children frequently model behaviors demonstrated by authority figures or salient characters, particularly when behaviors are paired with clear consequences. This theoretical lens is especially relevant in media-based safety instruction, where animated characters explicitly model both unsafe and corrective behaviors, allowing children to internalize safety norms through guided observation. Complementing this perspective, Ecological Systems Theory (16) situates safety learning within nested environmental systems, including the child’s immediate microsystem (home, school), mesosystem (home–school interactions), exosystem (community influences), and macrosystem (cultural values and policies). Safety education, therefore, cannot be viewed solely as classroom instruction but must be understood as a socially embedded process shaped by family practices, institutional policies, and broader cultural expectations. This ecological perspective underscores the importance of contextual alignment and reinforces the need for safety frameworks that integrate school, family, and community dynamics. Developmentally, early-childhood safety instruction must align with children’s cognitive and socio-emotional capacities. Constructivist developmental theory, drawing from (17, 18), emphasizes that children aged 3–6 operate largely within the preoperational stage, relying on concrete representations, visual cues, repetition, and guided scaffolding. Vygotsky’s concept of the Zone of Proximal Development further highlights the role of adult mediation and structured support in facilitating learning beyond children’s independent capabilities. These principles justify the use of narrative-based, visually rich, and scaffolded instructional approaches in safety education. From a behavioral perspective, Behavioral Skills Training (BST) offers an evidence-based instructional model widely used in injury prevention and safety interventions (15). BST consists of four components—instruction, modeling, rehearsal, and feedback—which together support skill acquisition and behavioral generalization. Numerous safety education programs have demonstrated the effectiveness of this structured approach in teaching children how to respond to hazards in real-life contexts. The incorporation of modeling and guided practice in safety pedagogy is therefore theoretically supported and empirically validated. Finally, Experiential Learning Theory (19) provides a process-oriented framework for understanding how children learn from concrete experiences, reflection, conceptualization, and active experimentation. Safety education often requires children to engage in simulated drills, role-playing activities, and reflective discussions that move beyond passive knowledge acquisition toward experiential understanding. This cyclical learning process reinforces retention and facilitates the transfer of safety skills across contexts. In sum, these theoretical foundations establish a coherent conceptual basis for multidimensional safety education in early childhood. They explain why safety learning must integrate modeling, contextual awareness, developmental alignment, structured behavioral rehearsal, and experiential engagement. Grounding safety education within these frameworks ensures that instructional design is not only empirically informed but also theoretically robust, thereby providing a strong foundation for the subsequent development of the Multidimensional Pedagogical Safety Integration Model (MPSIM).

## Method

### Study design overview

The present study employed a mixed qualitative research design, combining a Systematic Literature Review (SLR) with qualitative content analysis, followed by framework construction. The SLR was used to identify, synthesize, and categorize existing research on early-childhood safety education, ensuring methodological transparency and reproducibility following PRISMA guidelines (44). This phase enabled the extraction of multidimensional domains of safety education, including content, pedagogical, contextual, developmental, and implementation dimensions. Subsequently, qualitative content analysis (45) was applied to episodes of the animated series *Sheriff Labrador* to examine how safety content is represented within educational media. The combination of these methods provided both breadth and depth—the SLR established the theoretical foundation and evidence base, while content analysis yielded applied insights into real-world media representation. Finally, insights from both strands were integrated inductively to construct a process-based pedagogical framework, aligning with methodological approaches to model development in social sciences (43). This triangulated approach ensured that the resulting model was both empirically grounded and pedagogically relevant.

### Systematic literature review

This review was conducted following the PRISMA 2020 statement for systematic reviews. The methodology involved a systematic search of academic databases, a multi-stage screening process, and a qualitative synthesis of the 50 included studies.

#### Search strategy and databases

A comprehensive literature search was conducted on Google Scholar, Scopus (for open-access and indexed articles), and PubMed/PMC to identify relevant studies. The search was limited to peer-reviewed articles published between January 1980 and October 2024. Search terms included combinations of “early childhood safety education,” “preschool safety skills,” “injury prevention education,” and specific safety domains such as “fire safety,” “water safety,” and “pedestrian safety.”

#### Inclusion and exclusion criteria

Studies were selected based on a predefined set of inclusion and exclusion criteria. Inclusion criteria required articles to be peer-reviewed, written in English, focused on children aged 0–8, and centered on safety education interventions, curricula, or dimensions. Exclusion criteria eliminated grey literature, opinion pieces, studies on older children, research without an educational component, and articles not available in full text. A detailed breakdown of these criteria and selected references are available in the Supplementary materials S1, S2.

#### Study selection and data extraction

**Figure 1:**
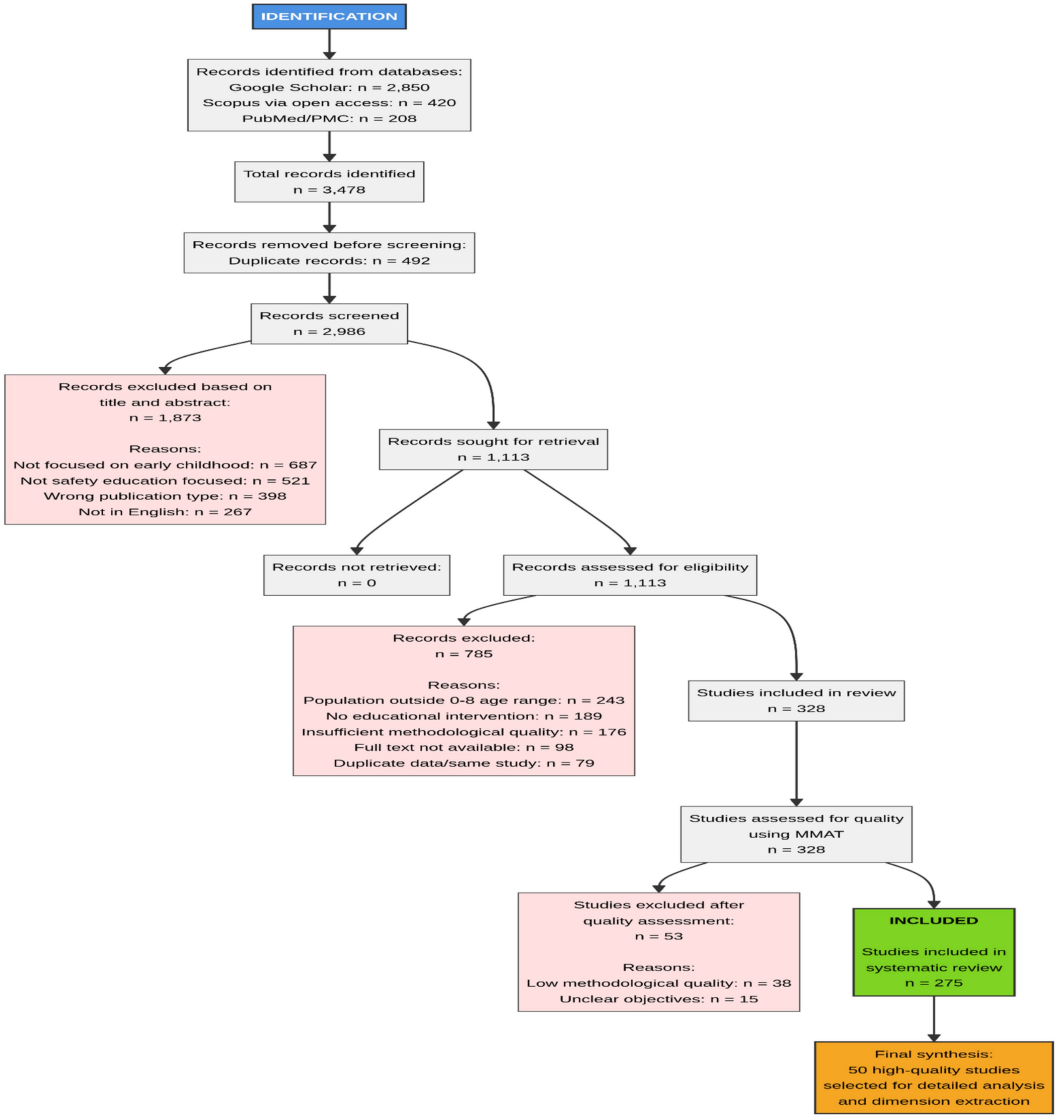
PRISMA 2020 flow diagram for the systematic review of early-childhood safety education studies.

The study selection process is illustrated in the PRISMA 2020 flowchart (Figure 1). Initial database searches identified 3,478 records. After removing duplicates, 2,986 records were screened by title and abstract, resulting in 1,113 articles for full-text eligibility assessment. Following a detailed review, 328 studies were included. These were further assessed for methodological quality using the Mixed Methods Appraisal Tool (MMAT), leading to a final synthesis of 50 high-quality studies that were used for data extraction and analysis.

#### Data extraction

Once the final set of 50 studies was identified, data were systematically extracted using a standardized extraction form to ensure consistency and comparability across sources. For each study, bibliographic details such as author, year of publication, and country of origin were recorded, followed by key study characteristics including research design (e.g., experimental, qualitative, review), sample size, and participant age range. The extraction process also captured the core findings relevant to early-childhood safety education, with attention to how each study addressed the five multidimensional domains. This included documenting the specific safety topics taught, the pedagogical strategies employed, contextual and cultural considerations, developmental adaptations used to tailor content to young learners, and implementation approaches such as program structure, training requirements, and reinforcement methods. This structured extraction process enabled a comprehensive synthesis of evidence across diverse studies and facilitated the identification of patterns, gaps, and converging themes that informed model development.

### Data synthesis: thematic analysis

To synthesize the extracted data and identify the core dimensions of early childhood safety education, a **thematic analysis** was conducted. This method was chosen for its flexibility and its suitability for identifying, analyzing, and reporting patterns (themes) within qualitative data. The process was guided by the six-phase framework (46), which ensured a rigorous and systematic approach.

### Coding

The first stage of the analysis consisted of an intensive coding process in which the *Results* and *Discussion* sections of all 50 included studies were examined line-by-line to identify any segment of text describing an aspect of early-childhood safety education. Each meaningful segment was assigned a descriptive code that captured its core idea, following an inductive and data-driven approach in which codes emerged directly from the content rather than from predefined categories. This open-coding stage produced a wide range of initial codes reflecting diverse facets of safety education, including examples such as “teaching firearm safety,” “stranger awareness rules,” “using role-play to practice skills,” “adapting lessons for preschoolers,” “parental involvement in safety training,” and “fire drill procedures.” This detailed and iterative coding process provided the foundation for grouping related codes, identifying thematic patterns, and ultimately synthesizing the multidimensional domains that informed subsequent stages of the analysis.

### Theme development

Theme development proceeded through a structured, hierarchical process that transformed the initial inductive codes into the five major dimensions of early-childhood safety education. First, similar codes were grouped into lower-level sub-dimensions; for example, the codes “teaching firearm safety,” “poison prevention skills,” and “playground hazard identification” were collated to form the sub-dimension *Physical Environment Hazards*. Next, related sub-dimensions were clustered into broader primary dimensions, such as grouping *Physical Environment Hazards*, *Traffic and Pedestrian Skills*, and *Water Safety Awareness* to create the initial primary dimension *Physical Environment & Community Safety*. In the final stage, all primary dimensions were reviewed and organized into five overarching categories that captured the foundational facets of safety education. The Content Dimension encompassed themes describing *what* is taught (e.g., physical safety, personal safety, health and hygiene), while the Pedagogical Dimension included themes addressing *how* safety is taught (e.g., learning methods, assessment strategies). The Contextual Dimension incorporated themes reflecting *where and under what circumstances* learning occurs (e.g., cultural influences, family involvement), and the Developmental Dimension represented themes concerning *for whom* safety instruction is designed (e.g., cognitive and socio-emotional adaptations). Finally, the Implementation Dimension captured systemic considerations related to *how safety learning is supported and sustained* (e.g., resource allocation, program quality, generalization).

### Thematic content analysis of *Sheriff Labrador*

#### Dataset: episodes

The dataset consisted of multiple episodes from the animated educational series *Sheriff Labrador*, which centers on safety education for young children. A total of 35 episodes were selected purposively, ensuring broad representation across safety-related storylines such as fire, traffic, water, hygiene, and personal protection. Each episode was treated as a unit of analysis and further segmented into meaningful scenes that depicted safety-related interactions between characters. The analysis focused on identifying and categorizing scenes that explicitly or implicitly communicated safety messages to preschool-aged viewers.

The selection of *Sheriff Labrador* was purposive rather than convenience-based, guided by its systematic coverage of safety themes, its structured modeling of corrective behaviors, and its suitability for deductive mapping onto the SLR-derived safety taxonomy.

#### Coding focus: content dimensions

The analysis focused exclusively on the Content Dimension of early-childhood safety education identified in the systematic review, applying seven major categories: Physical Environment Safety, Traffic and Pedestrian Safety, Water Safety, Personal Safety and Protection, Emergency Preparedness, Health and Hygiene Safety, and Rural and Agricultural Safety. Each category comprised multiple sub-dimensions, such as fire prevention, road awareness, drowning prevention, hygiene habits, and animal safety. All scenes were examined for both manifest content—explicit visual or verbal demonstrations of safety behaviors—and latent content, which included implied lessons or moral messages embedded within the storyline. For example, in *Fire at the Duck’s House*, manifest content illustrated the step-by-step fire evacuation procedure, whereas latent content reinforced the importance of emotional regulation, calmness, and teamwork during emergencies. This dual-layered coding approach allowed the analysis to capture not only the overt safety instruction provided in the series but also the implicit pedagogical cues designed to shape children’s attitudes, awareness, and decision-making in safety-related contexts.

#### Codebook derivation

The codebook was developed deductively from the five-dimensional framework generated during the Systematic Literature Review (SLR), with specific emphasis on the content dimension taxonomy. Each sub-dimension identified in the SLR (e.g., physical, water, health, personal, and rural safety) was operationalized into a coding category with definitions, examples, and decision rules. The initial codebook was pilot-tested on three randomly selected episodes to refine category boundaries and ensure conceptual clarity. Minor adjustments were made to merge overlapping codes and ensure alignment with established early-childhood safety constructs identified in prior research (1, 2).

#### Trustworthiness

To ensure trustworthiness, the study followed qualitative rigor criteria of credibility, dependability, confirmability, and transferability (47). Credibility was enhanced through iterative coding and peer debriefing with two researchers experienced in early-childhood safety pedagogy, who reviewed and validated the alignment between codes and corresponding scenes. Dependability was maintained by keeping a detailed audit trail documenting all coding decisions, category refinements, and analytical justifications throughout the process. Confirmability was strengthened through triangulation of manifest and latent content, ensuring that interpretations remained grounded in observable or clearly inferable scene elements rather than researcher bias. Transferability was supported by providing thick descriptions of episodes and scene contexts, enabling other researchers and educators to determine the relevance of findings to comparable media-based educational environments. Together, these procedures ensured that the thematic structure accurately reflected the depth and breadth of *Sheriff Labrador*’s representation of safety concepts and provided a robust empirical foundation for constructing the subsequent process-based pedagogical model.

#### Method of model development

The development of the Multidimensional Pedagogical Safety Integration Model (MPSIM) followed a rigorous, multi-step process that integrated evidence from the systematic literature review, thematic content analysis of *Sheriff Labrador*, and established learning theories. First, the five key domains of early-childhood safety education—Content, Pedagogical, Contextual, Developmental, and Implementation—were extracted from the SLR to form the foundational structure of the model. Next, findings from the content analysis were systematically aligned with these domains, allowing the identification of how empirically observed safety messages in the series corresponded to research-derived categories. A cross-dimensional integration phase then linked each content domain to appropriate pedagogical strategies, contextual considerations, developmental requirements, and implementation mechanisms, establishing a coherent matrix of relationships across the five dimensions. The model was theoretically grounded in Social Learning Theory (14), Ecological Systems Theory (16), Constructivist Developmental Theory (18), Behavioral Skills Training (15), and Experiential Learning Theory (19), ensuring developmental appropriateness, contextual sensitivity, and instructional validity. Through iterative synthesis, these dimensions were consolidated into a unified, process-based framework that traces the progression of safety learning from content selection to pedagogical delivery, contextual adaptation, developmental alignment, and sustained implementation. The preliminary model underwent expert review for clarity, coherence, and practical relevance, after which a universal teacher guide was developed to translate the theoretical framework into actionable instructional steps for classroom use.

## Results

### Results for research objective 1: multidimensional domains of early-childhood safety education

The systematic literature review identified five major dimensions of early-childhood safety education, comprising a total of 28 sub-dimensions drawn from the included studies. Table 1 shows five overarching dimensions were: Content, Pedagogical, Contextual, Developmental, and Implementation. Among these, the Content Dimension was the most extensively represented, with 7 sub-dimensions, followed by the Pedagogical Dimension (4 sub-dimensions), the Contextual Dimension (4 sub-dimensions), the Developmental Dimension (3 sub-dimensions), and the Implementation Dimension (3 sub-dimensions).

**Table 1:** Multidimensional domains of early-childhood safety education.

Dimension	No. of sub-dimensions	Example sub-dimensions
Content	7	Physical environment safety, traffic/pedestrian safety, water safety, personal safety, emergency preparedness, health & hygiene, rural/agricultural safety
Pedagogical	4	Integration approaches, learning methods (BST, modeling), assessment methods, professional development
Contextual	4	Cultural/socioeconomic influences, family/community involvement, regulatory frameworks, technological innovations
Developmental	3	Cognitive, physical, and social–emotional considerations
Implementation	3	Resource allocation, program standards, skill generalization/maintenance

Frequency of citations across the literature the content dimension received the highest number of supporting studies (e.g., 14 + distinct references across sub-dimensions), indicating strong consensus on the importance of domain-specific safety knowledge for young children. Pedagogical dimensions were supported by at least 10 references, highlighting the centrality of methods such as Behavioral Skills Training (BST), modeling, and in-situ assessment. Contextual and developmental dimensions were moderately represented (6–8 references each), reflecting the need to adapt safety education to family, cultural, and developmental factors. Implementation dimensions had fewer but still meaningful citations, emphasizing the importance of structural support, high-quality programming, and reinforcement mechanisms. Overall, the SLR demonstrates that early-childhood safety education is inherently multidimensional, extending beyond isolated safety topics to include pedagogy, developmental appropriateness, contextual alignment, and implementation quality. The strong representation of content and pedagogical dimensions suggests that safety education is most effective when grounded in specific risk domains and delivered through evidence-based instructional strategies such as modeling, role-play, and in-situ assessment. The presence of contextual and developmental dimensions underscores the importance of cultural responsiveness and age-appropriate design, while implementation dimensions highlight the structural and institutional requirements needed to sustain effective safety practices.

### Results for research objective 2: representation of safety content in *Sheriff Labrador*

The thematic content analysis examined how the Content Dimension of early-childhood safety education is represented across episodes of *Sheriff Labrador*. Using the seven pre-defined sub-dimensions (derived from the SLR), the analysis identified both manifest representations—explicit depictions of hazards, risk behaviors, and safety rules—and latent representations, which conveyed safety norms, emotional learning, and underlying protective principles. In total, the dataset as shown in Table 2 included 35 safety-focused episodes spanning multiple seasons, all of which contained at least one safety sub-dimension.

**Table 2:** A quantitative count of category appearances across episodes shows the distribution of safety content.

Safety sub-dimension	Number of episodes represented	Dominant representation type
Health & hygiene safety	8	Strong manifest + latent
Emergency preparedness	6	Strong manifest
Rural & agricultural safety	6	Moderate manifest, high latent
Physical environment safety	5	Predominantly manifest
Water safety	5	Predominantly manifest
Traffic & pedestrian safety	3	Manifest
Personal safety & protection	3	Latent + situational manifest

Health & Hygiene Safety was the most represented domain, while Traffic/Pedestrian Safety and Personal Protection appeared least frequently.

### Safety dimension physical environment safety

Episodes depicting physical environment safety illustrated a range of everyday hazards, including home fires, playground dangers, open manholes, escalator misuse, and unsafe street play. Manifest content featured explicit demonstrations of risky behaviors—such as standing too close to swings, playing near uncovered manholes, or misusing escalators—followed by corrective safety actions modeled step-by-step by *Sheriff Labrador*. Latent lessons embedded within these scenes emphasized the importance of vigilance, recognizing environmental risks, and reporting hazards to trusted adults. Episodes such as *Fire at the Duck’s House* and *Monsters Under the Manhole Cover* exemplified how visible incident scenarios were paired with moral messaging that encouraged responsibility, awareness, and proactive safety behavior.

### Traffic and pedestrian safety

Traffic and pedestrian safety were represented through scenes addressing unsafe walking behaviors, incorrect street-crossing practices, improper bus-riding routines, and distractions caused by digital devices near roadways. Manifest content included demonstrations of looking both ways before crossing, holding handrails inside buses, and following bus-safety rules. Meanwhile, latent content reinforced the broader principles of alertness, avoiding distractions, and making responsible pedestrian decisions. Episodes such as *Kids Learn School Bus Safety Tips* and *Focus on the Road* portrayed realistic traffic scenarios, helping children understand both the procedural and attitudinal components of road safety.

### Water safety

Water safety was portrayed across diverse scenarios such as flood dangers, swimming pool hazards, polluted open water, and unsafe water games. Manifest content delivered concrete safety rules—for example, warnings against walking through flood water, running near pools, or entering contaminated water—alongside demonstrations of proper safety actions. Latent content emphasized the roles of supervision, calm decision-making, and situational awareness near water. Episodes such as *Flood Safety*, *Swimming Pool Safety*, and *Water Pollution Rescue Mission* illustrated how the series combined visible safety instruction with emotional and cognitive lessons related to caution, self-regulation, and responsible water behavior.

### Personal safety and protection

Personal safety and protection themes focused on stranger danger, abduction prevention, and home door safety. Manifest representations included scenes featuring suspicious strangers, unsafe door-answering behaviors, and demonstrations of safe responses—such as not opening the door alone or seeking help when feeling followed. Latent content conveyed emotional and social lessons related to fear management, identifying trustworthy adults, and knowing when to seek support. Episodes like *Do not Trust Strangers* and *Who’s Following Me?* Highlighted both procedural knowledge and psychological readiness required for personal safety.

### Emergency preparedness

Emergency preparedness was represented through episodes addressing fire evacuation, basic first aid, earthquake response, flood evacuation, and communication with emergency services. Manifest scenes often depicted detailed evacuation sequences, first-aid steps, and demonstrations of dialing emergency numbers. Latent content reinforced core emergency principles such as staying calm, listening to instructions, cooperating with others, and understanding the role of community helpers. Episodes like *Fire at the Duck’s House* and *Call 911* exemplified how the series teaches both practical emergency responses and the emotional regulation necessary during crisis situations.

### Health and hygiene safety

Health and hygiene safety was the most frequently represented category, covering hygiene routines, cough etiquette, nutrition, oral hygiene, sleep habits, emotional safety, and physical activity. Manifest content featured clear demonstrations of handwashing, proper tooth-brushing, healthy eating choices, and active play, providing direct behavioral models for young children. Latent messages emphasized well-being, self-care, emotional regulation, and the development of healthy habits. Episodes such as *Hand-Washing Safety*, *Healthy Eating*, and *Do not Be Afraid* highlighted the series’ strong focus on holistic health and daily routines that build lifelong wellness practices.

### Rural and agricultural safety

Rural and agricultural safety was portrayed through scenarios involving wildlife encounters, dangerous farming tools, uneven outdoor terrain, and hazards such as bees, snakes, and rural water embankments. Manifest content showed unsafe interactions—approaching wild animals, mishandling tools, or playing near steep terrain—along with appropriate corrective behaviors modeled by *Sheriff Labrador*. Latent content stressed respect for nature, environmental caution, and awareness of rural risks that differ from urban environments. Episodes such as *Wild Animal on the Loose* and *Bee Sting Safety* demonstrated how the series uses rural settings to reinforce safety awareness in diverse ecological contexts.

Across episodes, *Sheriff Labrador* presents safety themes through a blend of explicit instruction (manifest) and embedded moral or protective meaning (latent). The series shows strong alignment with the SLR-derived content taxonomy, covering all seven safety sub-dimensions. However, representations are uneven, with disproportionately high focus on hygiene and emergency preparedness compared with traffic and personal protection. These patterns reflect a tendency to emphasize daily living safety and health routines, which are more easily represented in animated narrative forms. The findings confirm that *Sheriff Labrador* provides a rich content base for integrating media into early-childhood safety education pedagogies.

### Results for research objective 3: development of the multidimensional pedagogical safety integration model (MPSIM)

The synthesis of findings from the systematic literature review and the thematic content analysis resulted in the construction of the Multidimensional Pedagogical Safety Integration Model (MPSIM)—a unified framework designed to support the delivery of early-childhood safety education through structured, developmentally aligned, and contextually adaptive processes.

### Operationalization of the five dimensions

**Figure 2:**
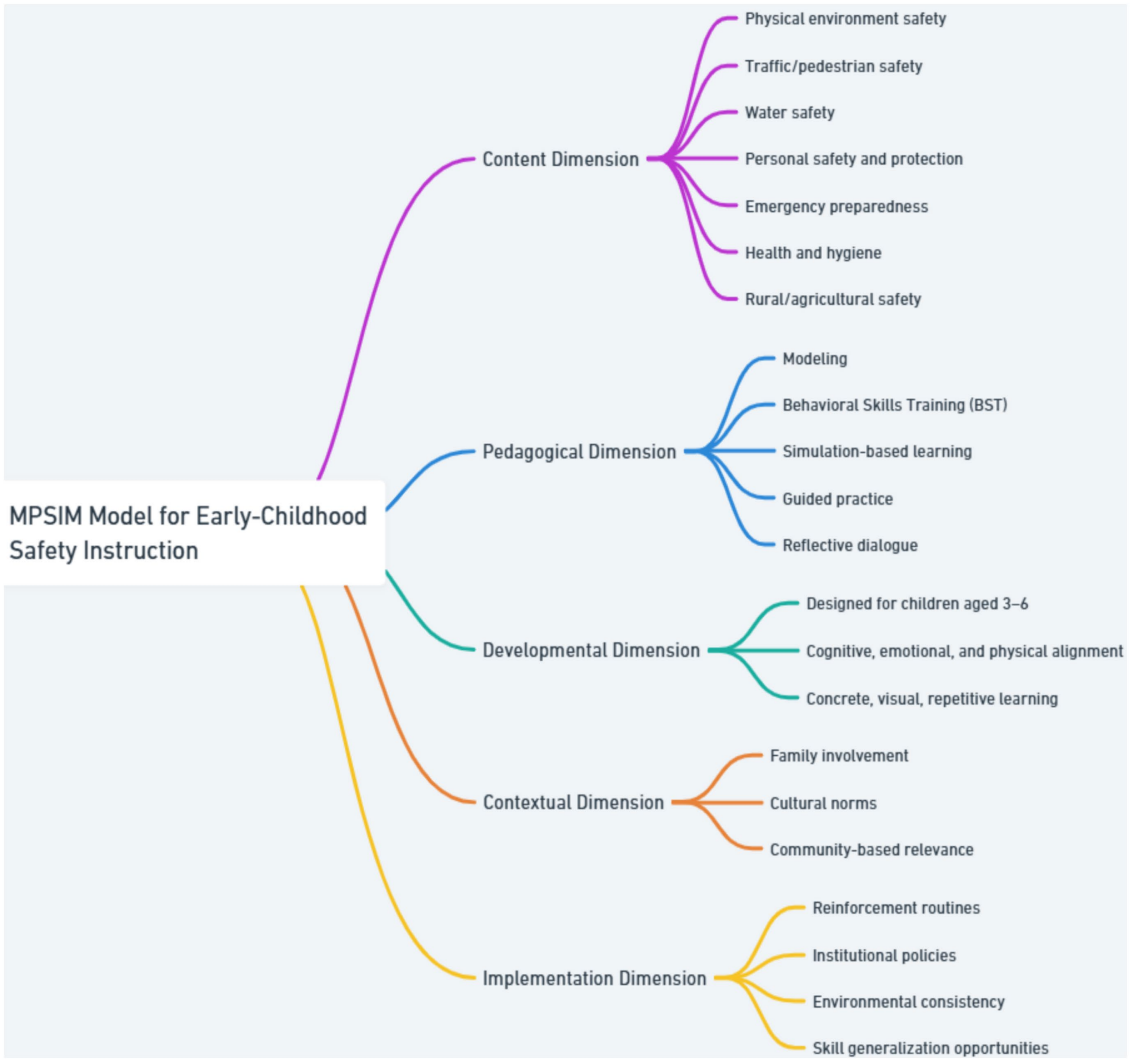
Multidimensional pedagogical safety integration model (MPSIM).

As shown in Figure 2, the Multidimensional Pedagogical Safety Integration Model (MPSIM) organizes early-childhood safety instruction through five interconnected phases that ensure comprehensive coverage, developmental appropriateness, and long-term retention of safety skills. The Content Dimension defines *what* children should learn, based on seven empirically derived safety sub-domains—physical environment safety, traffic/pedestrian safety, water safety, personal safety and protection, emergency preparedness, health and hygiene, and rural/agricultural safety—identified across the 35 analyzed episodes of *Sheriff Labrador*. These categories reflect the full spectrum of safety risks relevant to young children. The Pedagogical Dimension specifies *how* safety skills are taught using evidence-based instructional strategies including modeling, Behavioral Skills Training (BST), simulation-based learning, guided practice, and reflective dialogue. The Developmental Dimension addresses *for whom* instruction is designed, ensuring that safety content and delivery methods align with the cognitive, emotional, and physical capacities of children aged 3–6, who require concrete, visual, and repetitive learning experiences. The Contextual Dimension clarifies *under what conditions* safety learning occurs, emphasizing the significance of family involvement, cultural norms, and community-based relevance in shaping children’s comprehension and application of safety rules. Finally, the Implementation Dimension focuses on *how to sustain learning* through reinforcement routines, institutional policies, environmental consistency, and structured opportunities for skill generalization both inside and outside the classroom.

### Integration of media evidence with pedagogical theory

The integration of thematic content from *Sheriff Labrador* with established pedagogical theories reinforces the theoretical robustness of the MPSIM model. The series demonstrates clear alignment with Social Learning Theory (14), as episodes frequently depict characters modeling both safe and unsafe behaviors, allowing children to observe consequences and internalize appropriate responses through vicarious learning. This strong representational pattern supports the use of modeling and guided observation as core teaching strategies within the model. Additionally, Ecological Systems Theory provides the contextual foundation by situating safety learning within children’s nested environments: microsystems (home, school), mesosystems (family–school interaction), and macrosystems (cultural and societal expectations about safety) (16). The Constructivist perspectives further justify the emphasis on developmental alignment, as the content and tasks are structured to match young children’s need for concrete representation, scaffolding, and hands-on engagement (17). Meanwhile, Behavioral Skills Training (BST) supports the iterative teaching cycle—instruction, modeling, rehearsal, and feedback—reflected in both media content and recommended teaching strategies (15). Finally, Experiential Learning Theory underpins the inclusion of practice-based simulations, guided reflection, and real-world application, ensuring that safety concepts are not only learned but also transferred across contexts (19). Together, these theoretical pillars validate the integration of media-based evidence with pedagogically sound instructional design.

## Discussion

This study examined how the multidimensional nature of early-childhood safety education, identified through a systematic literature review, aligns with safety representations in the children’s animated series *Sheriff Labrador*, and how these findings informed the development of the Multidimensional Pedagogical Safety Integration Model (MPSIM). The discussion integrates theoretical perspectives, empirical findings, and practical implications for early-childhood educators.

These findings situate *Sheriff Labrador* within the broader lineage of educational television and entertainment–education media, which have historically demonstrated that structured narrative modeling can facilitate both knowledge acquisition and behavioral internalization among young audiences (38, 41).

Moreover, the findings demonstrate strong convergence between the seven content sub-domains identified in the SLR—physical environment safety, traffic/pedestrian safety, water safety, personal safety and protection, emergency preparedness, health and hygiene, and rural/agricultural safety—and the themes represented across the 35 analyzed episodes of *Sheriff Labrador*. This overlap suggests that the series provides a substantive representation of real-world hazards commonly addressed in early-childhood safety literature (1, 3). The mapping further confirms that animated media can encapsulate multidimensional safety concepts grounded in empirical research, demonstrating their potential as complementary instructional resources in early-childhood education.

Although all seven domains appeared across episodes, their representation was uneven. Health and hygiene safety and emergency preparedness emerged as the most frequently depicted categories, echoing global educational trends that emphasize disease prevention and emergency response in early childhood (5). Conversely, traffic and pedestrian safety and personal safety/protection appeared less frequently, despite being commonly addressed in safety education programs (2, 10). This imbalance suggests that while the series offers a broad safety foundation, teachers may need to supplement less-represented domains with additional resources.

The findings underscore *Sheriff Labrador*’s strong alignment with Social Learning Theory, particularly the processes of modeling, observational learning, and vicarious reinforcement (14). Many episodes explicitly demonstrate unsafe behavior, followed by corrective modeling from *Sheriff Labrador*, mirroring the recommended sequence for effective safety instruction (22). Such patterns also align with Behavioral Skills Training (BST)—instruction, modeling, rehearsal, and feedback—which is widely supported in the safety education literature (15). The show’s use of narrative consequences and problem-solving further reinforces children’s internalization of safety norms through visual and emotional cues, indicating that animated media can serve as a powerful instructional scaffold.

The safety messages embedded in the episodes align well with the developmental needs of children aged 3–6, an age group situated within Piaget’s preoperational stage, characterized by reliance on visual cues, concrete examples, and simple narratives (17). The show’s repetitive structure, emotional expression, and cause-and-effect sequencing complement young children’s learning capacities, reflecting principles of constructivist developmental theory (18). Additionally, the emotional framing of fear, danger, reassurance, and helping behaviors supports socio-emotional learning, an essential component of developmental safety (48). These findings affirm that *Sheriff Labrador* provides developmentally appropriate representations that align with cognitive and emotional scaffolding recommended in early-childhood pedagogy.

The contextual analysis shows that many of the safety scenarios in the series—such as home fires, street hazards, playground accidents, and hygiene routines—are culturally neutral and universally relevant. This aligns with Bronfenbrenner’s Ecological Systems Theory, which highlights the influence of home, school, and community environments in shaping children’s learning (16). Episodes involving family members, community helpers, and school settings demonstrate how safety behaviors are embedded within microsystems and mesosystems. Furthermore, the inclusion of agricultural and rural safety themes reflects broader macrosystemic influences, making the series adaptable to diverse cultural and socioeconomic contexts (49).

By synthesizing SLR insights and media-based evidence, this study produced the Multidimensional Pedagogical Safety Integration Model (MPSIM). The model unifies five essential dimensions—Content, Pedagogical, Developmental, Contextual, and Implementation—into a dynamic safety instruction process. The model’s layering reflects the complexity of safety education identified in the literature (7, 8). MPSIM’s theoretical grounding creates a strong foundation for early-childhood safety education, integrating modeling (Bandura), contextual systems (16), developmental alignment (17, 18), experiential cycles (19), and behavior-based instruction (15). This multidimensional synthesis responds directly to prior concerns about the lack of structured, evidence-based frameworks in early childhood safety education (14).

The findings suggest opportunities for integrating media-based safety instruction into early-childhood curricula, particularly where safety education is fragmented or underdeveloped (21). Policymakers may also draw upon MPSIM to establish guidelines for using animation and child-friendly media as structured instructional tools. Such strategies may support more consistent implementation of safety routines across school systems and foster stronger collaboration between families, communities, and early-childhood educators.

This study makes several important contributions to early-childhood safety education. It is among the first to systematically map an animated children’s series onto empirically derived safety education dimensions, demonstrating how multimedia narratives can align with research-based safety domains. It also provides one of the earliest evaluations of *Sheriff Labrador* as an instructional medium, highlighting its potential for modeling safe behaviors and reinforcing conceptual understanding. By integrating systematic literature review findings with media analysis, the study contributes a comprehensive, theory-informed pedagogical model that synthesizes content, pedagogical, developmental, contextual, and implementation dimensions. In addition, it introduces a universal teacher guide that operationalizes this model through developmentally appropriate, pedagogically grounded, and context-sensitive instructional strategies. Collectively, these contributions address the widely documented gap in structured, evidence-based frameworks for early-childhood safety education and offer a practical foundation for improving safety instruction in early learning environments.

The Multidimensional Pedagogical Safety Integration Model (MPSIM) can be operationalized across classroom, curriculum, and policy levels through coordinated, structured actions aligned with its five dimensions. At the classroom level, teachers may implement a five-step instructional cycle: selecting a safety domain (e.g., fire safety), introducing a relevant *Sheriff Labrador* episode to model appropriate and inappropriate behaviors (Content and Pedagogical Dimensions), guiding children through discussion and Behavioral Skills Training sequences involving instruction, modeling, rehearsal, and feedback (Pedagogical Dimension), adapting activities to the cognitive and socio-emotional characteristics of 3–6-year-olds using visual scaffolds and simplified routines (Developmental Dimension), and reinforcing learning through recurring drills and home–school safety checklists (Implementation Dimension). At the curriculum level, designers can map the seven safety domains across the academic year, ensuring each unit specifies learning objectives, instructional strategies, contextual adaptations, developmental differentiation, and reinforcement mechanisms. At the policy level, educational authorities may establish tiered safety standards that define minimum content coverage, require teacher training aligned with evidence-based safety pedagogy, and institutionalize regular monitoring and community partnerships to sustain safety practices beyond isolated classroom interventions.

## Limitations and future directions

This study has several limitations that should be acknowledged. First, the thematic content analysis was limited to 35 episodes of *Sheriff Labrador*, which may not fully represent the entire scope of safety scenarios portrayed across the series. Although the selected episodes were purposively chosen to ensure broad thematic coverage, the possibility remains that additional safety domains or variations in representation exist beyond the analyzed dataset. Second, the analysis focused exclusively on media content representation and did not empirically assess children’s learning outcomes, behavioral change, or skill retention following exposure to the series or to instruction guided by the Multidimensional Pedagogical Safety Integration Model (MPSIM). Consequently, while the model is theoretically grounded and logically constructed, it has not yet been validated through classroom-based intervention studies, longitudinal implementation research, or controlled outcome evaluations. Empirical testing across diverse educational settings is necessary to determine its practical effectiveness and generalizability.

In addition to these methodological constraints, the successful application of MPSIM depends substantially on the professional competence, creativity, and multidisciplinary expertise of early-childhood educators. Although *Sheriff Labrador* provides structured safety narratives and modeled behaviors, media exposure alone does not ensure meaningful learning. The transformation of animated content into effective pedagogy requires teachers to exercise integrated knowledge across developmental psychology, health education, communication strategies, experiential learning design, and classroom management. Educators function not merely as facilitators of media viewing but as instructional designers who scaffold discussion, guide rehearsal, contextualize scenarios, and adapt safety instruction to children’s cognitive, emotional, and cultural realities. Thus, the model’s effectiveness is contingent upon teacher agency and innovative pedagogical mediation.

Furthermore, this study examined a single animated series as an illustrative case. While *Sheriff Labrador* demonstrated strong alignment with empirically derived safety domains, the proposed framework is not medium-specific. Future research should investigate the applicability of MPSIM across diverse instructional modalities, including interactive digital applications, story-based simulations, puppetry, augmented reality tools, community-based experiential programs, and other forms of educational media. Expanding the framework beyond animation will enhance its theoretical scalability and practical adaptability, enabling the continued development of innovative, multimodal approaches to early-childhood safety education.

## Conclusion

This study set out to address the critical need for structured, evidence-based approaches to early-childhood safety education by examining the multidimensional nature of safety domains, analyzing how these domains are represented in the animated series *Sheriff Labrador*, and constructing the Multidimensional Pedagogical Safety Integration Model (MPSIM) to guide classroom practice. The systematic literature review confirmed that early-childhood safety education is inherently multidimensional, encompassing content, pedagogical, developmental, contextual, and implementation considerations. This comprehensive foundation was essential for understanding the complexity of safety learning and the range of competencies children must acquire to navigate hazards in everyday environments.

The findings further demonstrated that *Sheriff Labrador* offers substantial alignment with the seven content dimensions identified in the literature, illustrating its relevance as an emerging instructional resource for early-childhood safety education. Although some domains—such as health and hygiene safety and emergency preparedness—were more prominently represented, the series nonetheless covers all major safety categories, showing strong potential as a pedagogical tool. Its consistent use of modeling, demonstration, corrective feedback, and emotionally engaging narratives aligns closely with Social Learning Theory and developmental learning principles, reinforcing its suitability for children aged 3–6.

A key contribution of this study is the development of the Multidimensional Pedagogical Safety Integration Model (MPSIM), which synthesizes empirical evidence from the SLR and media analysis with established learning theories. MPSIM offers a coherent, process-based framework that operationalizes safety education through five interconnected dimensions: what to teach (Content), how to teach it (Pedagogy), for whom (Developmental alignment), under what conditions (Context), and how to sustain learning (Implementation). This model directly addresses the long-recognized gap in structured safety education frameworks and provides educators with a practical, theoretically grounded approach for designing meaningful safety instruction.

Most importantly, this study directly addresses the central question of how children’s media can be integrated with structured instructional frameworks. The findings demonstrate that integration is achieved through a systematic alignment process: first, empirically derived safety domains from the systematic literature review define what must be taught; second, media representations in *Sheriff Labrador* are mapped onto these domains to identify instructional relevance; third, pedagogical strategies grounded in Social Learning Theory, Behavioral Skills Training, and Constructivist principles structure how the content is delivered; fourth, contextual and developmental considerations ensure appropriateness for young learners; and finally, implementation mechanisms sustain learning beyond isolated media exposure. Through this multidimensional alignment, media content is transformed from passive entertainment into an evidence-based instructional resource embedded within a coherent pedagogical framework.

Teachers can operationalize MPSIM through a five-step instructional cycle: (1) select a safety domain; (2) introduce media-based modeling; (3) conduct guided rehearsal using BST principles; (4) adapt tasks to developmental level; and (5) reinforce learning through structured routines and family engagement tools.

In conclusion, this study demonstrates that integrating research-based safety domains with high-quality children’s media and established learning theories can meaningfully advance early-childhood safety education. By offering a comprehensive pedagogical model and a practical teacher guide, it contributes a timely and necessary framework that supports educators in preparing young children to navigate their world safely and confidently.

## Data Availability

The original contributions presented in the study are included in the article/Supplementary material, further inquiries can be directed to the corresponding author.
