# Supplementary material for: Developing a multidimensional pedagogical framework for safety education in early childhood: mapping systematic review insights to Sheriff Labrador
Source: Front Public Health. 2026 Mar 13;14:1765782. doi: 10.3389/fpubh.2026.1765782 (PMC13021869; doi:10.3389/fpubh.2026.1765782)
Supplement: Supplementary file 1 [file Presentation_1.pdf]

(Supplementary Material-S1)-Detailed Inclusion and Exclusion Criteria

Inclusion Criteria

Publication Type:

- Peer-reviewed journal articles
- Systematic reviews and meta-analyses
- Empirical research studies (quantitative, qualitative, or mixed methods)

Population:

- Children aged 0-8 years (early childhood)
- Early childhood education and care (ECEC) settings
- Preschool, kindergarten, and early primary school children

Topic Focus:

- Safety education programs and curricula
- Safety skills training
- Injury prevention education
- Health and safety protocols in early childhood settings
- Dimensions, domains, or components of safety education

Language:

- Articles written in English

Methodology:

- Studies employing quantitative, qualitative, or mixed methods
- Intervention studies
- Observational studies
- Comparative studies

Exclusion Criteria

Publication Type:

- Grey literature (conference proceedings, dissertations, theses)
- Opinion pieces and editorials
- Non-peer-reviewed publications
- Book chapters without empirical data

Population:

- Studies focused solely on children outside the 0-8 age range

- Studies in primary or secondary education settings (children over 8 years)
- Adult-only populations

Topic Focus:

- Studies focused solely on physical safety of environments without educational component
- Medical treatment or clinical interventions without educational focus
- Studies on child abuse detection/reporting without prevention education component
- Workplace safety for educators (not child-focused)

Language:

- Articles not written in English (due to resource constraints)

Availability:

- Articles without full-text availability
- Abstracts only

(Supplementary Material-S2)- Citation Mapping for All 50 Selected Studies

#	Study	Primary Contribution	Dimension Category
[1]	Barr et al. (2009)	Overview of safety curriculum in early childhood	Content (Multiple), Context
[2]	World Health Organization (2008)	Child injury prevention framework	Content, Implementation
[3]	Baruni & Miltenberger (2022)	Teaching safety skills - critical features	Pedagogy, Assessment
[4]	Gatheridge et al. (2004)	Firearm injury prevention programs	Content (Physical Environment)
[5]	Dancho et al. (2008)	Teaching poison hazard avoidance	Content (Physical Environment)
[6]	Kendrick et al. (2008)	Poisoning prevention education	Content (Physical Environment)
[7]	Duperrex et al. (2002)	Pedestrian safety education	Content (Traffic Safety)
[8]	Schwebel et al. (2014)	Child pedestrian safety interventions	Content (Traffic Safety)
[9]	Beck & Miltenberger (2009)	Abduction prevention skills	Content (Personal Safety)
[10]	Miltenberger et al. (2020)	Child abduction prevention research	Content (Personal Safety)
[11]	Wurtele et al. (1989)	Personal safety skills programs	Content (Personal Safety)
[12]	Sarhngi Kadijani et al. (2020)	Dimensions of safety education in emergencies	Content (Emergency), Pedagogy
[13]	Chakraborty et al. (2025)	Health and safety protocols in ECE	Content (Health/Hygiene), Context, Implementation
[14]	Giannakakos et al. (2020)	Behavioral skills training review	Pedagogy (Learning Methods)
[15]	Miltenberger (2008)	Teaching safety skills overview	Pedagogy (Assessment)
[16]	Saltmarsh (2010)	Cultural politics in safety education	Context (Cultural)
[17]	Himle et al. (2004a)	Gun play prevention	Content (Physical Environment)
[18]	Himle et al. (2004b)	Gun play prevention procedures	Content (Physical Environment)
[19]	Kelso et al. (2007)	Gun play prevention comparison	Content (Physical Environment)

[20]	Kendrick et al. (2013)	Home safety education	Content (Physical Environment)
[21]	Lumley et al. (1998)	Sexual abuse prevention	Content (Personal Safety)
[22]	Miltenberger et al. (1999)	Sexual abuse prevention training	Content (Personal Safety)
[23]	Miltenberger et al. (2004)	Behavioral skills training for gun safety	Pedagogy (Learning Methods)
[24]	Miltenberger et al. (2005)	In situ training evaluation	Pedagogy (Assessment)
[25]	Miltenberger et al. (2009)	BST with simulated in situ training	Pedagogy (Learning Methods)
[26]	Miltenberger et al. (1990)	Parent vs expert instruction	Pedagogy, Context (Family)
[27]	Mulvaney et al. (2011)	Safety education impact review	Content (Multiple), Implementation
[28]	Pac (2021)	ECEC access and child safety	Implementation, Context
[29]	Poche et al. (1981)	Teaching self-protection	Content (Personal Safety)
[30]	Summers et al. (2011)	Household safety for children with autism	Content (Physical Environment), Development
[31]	Crumrine, D. (2006)	Safety skills for autism spectrum disorder	Development, Pedagogy
[32]	Zuzeviciute (2012)	Dimensions of safety in educational settings	Content (Multiple), Implementation
[33]	Allen et al. (2020)	School belonging and safety	Context (Social-Emotional)
[34]	Beltman et al. (2011)	Teacher resilience	Context (Professional Development)
[35]	Greszler & Burke, (2022)	Early childhood education post-pandemic	Context, Implementation
[36]	De Stasio et al. (2019)	Children's emotion understanding	Development (Social-Emotional)
[37]	Fang et al. (2023)	Physical vs virtual activities	Content (Physical Environment)

[38]	Forgeard & Seligman (2012)	Optimism and well-being	Development (Social-Emotional)
[39]	Muir & Strnadová, (2014)	Resilience and responsibility	Context (Family)
[40]	Lehtisalo et al. (2010)	Dietary patterns and nutrition	Content (Health/Hygiene)
[41]	Harris (2017)	Work of imagination in development	Development (Cognitive)
[42]	Howitt & Jobling (2021)	Science education in early years	Pedagogy (Integration)
[43]	Kaplan (1995)	Quality of life and resource allocation	Implementation (Resources)
[44]	Badenoch & Bogdan, (2012)	Neurobiology of resilience	Development (Social-Emotional)
[45]	Randall et al. (2021)	Parental pressure and adjustment	Context (Family)
[46]	Shokoohi et al., (2012)	Socio-economic determinants of health	Context (Socioeconomic)
[47]	Bradshaw, W. (2013)	Cultural competence in ECE	Context (Cultural)
[48]	Randall & Jones, (1993)	Teaching fire safety skills	Content (Physical Environment)
[49]	Jerebine et al. (2022)	Children's voices in ECE safety	Context (Child Perspective)
[50]	WHO (2019)	Physical activity guidelines for young children	Content (Health/Hygiene)

RESULTS - Content Dimensions

3.1.1 Physical Environment Safety

- **Fire safety**: [1, 4, 48]
- **Burn prevention**: [17]
- **Poisoning prevention**: [5, 6, 30]
- **Firearm safety**: [4, 17, 18, 19, 23, 24, 25]
- **Home safety**: [20]
- **Playground safety**: [49]

3.1.2 Traffic & Pedestrian Safety

- **Pedestrian safety**: [7, 8]
- **Road safety**: [1]

3.1.3 Water Safety

- **Drowning prevention**: [1]

3.1.4 Personal Safety & Protection

- **Abduction prevention**: [9, 10, 29]
- **Stranger awareness**: [16]
- **Sexual abuse prevention**: [11, 21, 22]

3.1.5 Emergency Preparedness

- **Disaster education**: [12]
- **Emergency response**: [12]

3.1.6 Health & Hygiene Safety

- **Hygiene protocols**: [13]
- **Nutrition**: [40]
- **Mental health**: [13]
- **Physical activity**: [50]

3.1.7 Rural & Agricultural Safety

- **Farm safety**: [1]

RESULTS - Pedagogical Dimensions

3.2.1 Integration Approaches

- **Curriculum integration**: [12, 42]

3.2.2 Learning Methods

- **Behavioral Skills Training**: [3, 14, 23, 25]
- **Video modeling**: [9]
- **Simulation training**: [12, 25]

3.2.3 Assessment Methods

- **In situ assessment**: [3, 15, 24]

- **Multiple assessment types**: [3, 15]

3.2.4 Professional Development

- **Teacher training**: [12, 13, 34]
- **Parent instruction**: [26]

RESULTS - Contextual Dimensions

3.3.1 Cultural & Socioeconomic

- **Cultural responsiveness**: [13, 16, 47]
- **Socioeconomic factors**: [13, 46]

3.3.2 Family & Community

- **Parent engagement**: [13, 26, 39, 45]
- **Community resources**: [16]

3.3.3 Regulatory Frameworks

- **Standards and compliance**: [13]

3.3.4 Technological Innovations

- **Digital tools**: [13]
- **Virtual vs physical activities**: [37]

RESULTS - Developmental Dimensions

3.4.1 Cognitive Development

- **Concrete thinking**: [41]
- **Attention span considerations**: General developmental literature

3.4.2 Physical Development

- **Motor skills**: General developmental literature

3.4.3 Social-Emotional Development

- **Emotional regulation**: [13, 36, 38, 44]
- **Trust and attachment**: [13, 33]

RESULTS - Implementation Dimensions

3.5.1 Resource Allocation

- **Funding and resources**: [13, 43]

3.5.2 Program Quality

- **Evidence-based practices**: [3, 14, 27]
- **Quality standards**: [28, 32]

3.5.3 Generalization & Maintenance

- **Skill transfer**: [3, 15, 24]
- **Long-term retention**: [3]

References

- Barr, J., Saltmarsh, S., & Klopper, C. (2009). Early childhood safety education: An overview of safety curriculum and pedagogy in outer metropolitan, regional and rural NSW. *Australasian Journal of Early Childhood, 34*(4), 57–66.
- Baruni, R. R., & Miltenberger, R. G. (2022). Teaching safety skills to children: A discussion of critical features and practice recommendations. *Behavior Analysis in Practice, 15*(3), 938–950.
- Beck, K. V., & Miltenberger, R. G. (2009). Evaluation of a commercially available program and in situ training by parents to teach abduction-prevention skills to children. *Journal of Applied Behavior Analysis, 42*(4), 761–772.
- Chakraborty, D., Siddika, M. A., & Rahman, S. A. (2025). Health and safety protocols in early childhood education: Best practices and challenges. *Global Mainstream Journal of Health, Medicine & Hospitality Management, 4*(1), 1–18. https://doi.org/10.62304/jhmhm.v4i01.224
- Dancho, K. A., Thompson, R. H., & Rhoades, M. M. (2008). Teaching preschool children to avoid poison hazards. *Journal of Applied Behavior Analysis, 41*(2), 267–271.
- Duperrex, O., Roberts, I., & Bunn, F. (2002). Safety education of pedestrians for injury prevention: A systematic review of randomised controlled trials. *BMJ, 324*(7346), 1129.
- Gatheridge, B. J., Miltenberger, R. G., Huneke, D. F., Satterlund, M. J., Mattern, A. R., Johnson, B. M., & Flessner, C. A. (2004). Comparison of two programs to teach firearm injury prevention skills to 6- and 7-year-old children. *Pediatrics, 114*(3), e294–e299.
- Giannakakos, A. R., Vladescu, J. C., Reeve, K. F., Kisamore, A. N., Fienup, D. M., & Carrow, J. N. (2021). Using behavioral skills training and equivalence-based instruction to teach children safe responding to dangerous stimuli: A proof of concept. *The Psychological Record*, *71*(1), 119-131.
- Himle, M. B., Miltenberger, R. G., Flessner, C., & Gatheridge, B. (2004a). Teaching safety skills to children to prevent gun play. *Journal of Applied Behavior Analysis, 37*(1), 1–9.

- Himle, M. B., Miltenberger, R. G., Gatheridge, B. J., & Flessner, C. A. (2004b). An evaluation of two procedures for training skills to prevent gun play in children. *Pediatrics, 113*(1), 70–77.
- Randall, J., & Jones, R. T. (1993). Teaching children fire safety skills. *Fire Technology, 29*(3), 268–280.
- Jerebine, A., Fitton-Davies, K., Cortis, N., Cowley, F., & Katz, I. (2022). "All the fun stuff, the teachers say, 'that's dangerous!'": Hearing children's voices in early childhood education and care. *International Journal of Behavioral Nutrition and Physical Activity, 19*(1), 66.
- Kelso, P. D., Miltenberger, R. G., Waters, M. A., Egemo-Helm, K., & Bagne, A. G. (2007). Teaching skills to second and third grade children to prevent gun play: A comparison of procedures. *Education and Treatment of Children, 30*(3), 29–48.
- Kendrick, D., Smith, S., Sutton, A., Watson, M., Coupland, C., Mulvaney, C., & Mason-Jones, A. (2008). Effect of education and safety equipment on poisoning-prevention practices and poisoning: Systematic review, meta-analysis and meta-regression. *Archives of Disease in Childhood, 93*(7), 599–608.
- Kendrick, D., Young, B., Mason-Jones, A. J., Ilyas, N., Achana, F. A., Cooper, N. J., et al. (2013). Home safety education and provision of safety equipment for injury prevention. *Evidence-Based Child Health, 8*(3), 761–939.
- Lumley, V. A., Miltenberger, R. G., Long, E. S., Rapp, J. T., & Roberts, J. A. (1998). Evaluation of a sexual abuse prevention program for adults with mental retardation. *Journal of Applied Behavior Analysis, 31*(1), 91–101.
- Miltenberger, R. G. (2008). Teaching safety skills to children: Prevention of firearm injury, abduction, and sexual abuse. *ABA Newsletter, 31*(3), 1–4.
- Miltenberger, R. G., Flessner, C., Gatheridge, B., Johnson, B., Satterlund, M., & Egemo, K. (2004). Evaluation of behavioral skills training to prevent gun play in children. *Journal of Applied Behavior Analysis, 37*(4), 513–516.
- Miltenberger, R. G., Gatheridge, B. J., Satterlund, M., Egemo-Helm, K. R., Johnson, B. M., Jostad, C., et al. (2005). Teaching safety skills to children to prevent gun play: An evaluation of in situ training. *Journal of Applied Behavior Analysis, 38*(3), 395–398.
- Miltenberger, R. G., Gross, A., Knudson, P., Breitwieser, C., Bosch, A., Fuqua, R. W., & Jostad, C. (2009). Evaluating behavioral skills training with and without simulated in situ training for teaching safety skills to children. *Education and Treatment of Children, 32*(1), 63–75.
- Miltenberger, R. G., Thiesse-Duffy, E., Suda, K. T., Kozak, C., & Bruellman, J. (1990). Teaching prevention skills to children: The use of multiple measures to evaluate parent versus expert instruction. *Child & Family Behavior Therapy, 12*(4), 65–87.
- Miltenberger, R. G., Roberts, J. A., Ellingson, S., Galensky, T., Rapp, J. T., Long, E. S., & Lumley, V. A. (1999). Training and generalization of sexual abuse prevention skills for women with mental retardation. *Journal of Applied Behavior Analysis, 32*(3), 385–388.
- Miltenberger, R. G., Sanchez, S., & Valbuena, D. (2020). A review of behavioral prevention research on child abduction. *Journal of Applied Behavior Analysis, 53*(4), 1861–1876.
- Mulvaney, C. A., Watson, M. C., Errington, G., & Kendrick, D. (2011). Safety education impact and good practice: A review. *Health Education, 111*(5), 423–442.
- Pac, J. (2021). Early childhood education and care programs in the United States: Does access improve child safety? *Social Service Review, 95*(1), 97–135.

- Poche, C., Brouwer, R., & Swearingen, M. (1981). Teaching self-protection to young children. *Journal of Applied Behavior Analysis, 14*(2), 169–176.
- Saltmarsh, S. (2010). Lessons in safety: Cultural politics and safety education in a multiracial, multiethnic early childhood education setting. *Contemporary Issues in Early Childhood, 11*(3), 288–298.
- Sarhngi Kadijani, M., Araghie, A., Soshabi, P., Sadeghi, A., & Mahdizadeh, A. (2020). Identifying and explaining the dimensions of children's safety education in emergencies. *Journal of Police Medicine, 9*(4), 199–210.
- Schwebel, D. C., Barton, B. K., Shen, J., Wells, H. L., Bogar, A., Heath, G., & McCullough, D. (2014). Systematic review and meta-analysis of behavioral interventions to improve child pedestrian safety. *Journal of Pediatric Psychology, 39*(8), 826–845.
- Summers, J., Tarbox, J., Findel-Pyles, R. S., Wilke, A. E., Bergstrom, R., & Williams, W. L. (2011). Teaching two household safety skills to children with autism. *Research in Autism Spectrum Disorders, 5*(1), 629–632.
- Crumrine, D. (2006). *Teaching safety skills to children with autism spectrum disorders: A comparison of strategies* (Doctoral dissertation).
- Wurtele, S. K., Kast, L. C., Miller-Perrin, C. L., & Kondrick, P. A. (1989). Comparison of programs for teaching personal safety skills to preschoolers. *Journal of Consulting and Clinical Psychology, 57*(4), 505–511.
- Zuzeviciute, V. (2012). Dimensions of safety in educational settings: Lithuanian case. *International Journal of Education Law and Policy, 8*(1), 235–247.
- Allen, K. A., Vella-Brodrick, D., & Waters, L. (2020). Fostering school belonging in secondary schools using a socio-ecological framework. *Educational & Developmental Psychologist, 37*(1), 1–10.
- Beltman, S., Mansfield, C., & Price, A. (2011). Thriving not just surviving: A review of research on teacher resilience. *Educational Research Review, 6*(3), 185–207.
- Greszler, R., & Burke, L. M. (2020). Rethinking early childhood education and childcare in the COVID-19 era. *Backgrounder, No. 3533*. Heritage Foundation.
- De Stasio, S., Fiorilli, C., & Di Chiacchio, C. (2019). Effects of verbal ability and fluid intelligence on children's emotion understanding. *International Journal of Psychology, 54*(1), 47–53.
- Fang, W. T., Ng, E., & Chang, M. C. (2023). Physical outdoor activities versus virtual outdoor activities: Their different impacts on environmental behaviors. *International Journal of Environmental Research and Public Health, 20*(4), 3365.
- Forgeard, M. J., & Seligman, M. E. (2012). Seeing the glass half full: A review of the causes and consequences of optimism. *Pratiques Psychologiques, 18*(2), 107–120.
- Muir, K., & Strnadová, I. (2014). Whose responsibility? Resilience in families of children with developmental disabilities. *Disability & Society, 29*(6), 922–937.
- Lehtisalo, J., Erkkola, M., Tapanainen, H., Kronberg-Kippilä, C., Veijola, R., Knip, M., & Virtanen, S. M. (2010). Food consumption and nutrient intake in day care and at home in 3-year-old Finnish children. *Public Health Nutrition, 13*(6A), 957–964.
- Harris, P. L. (2017). *The work of the imagination*. John Wiley & Sons.
- Howitt, C., & Jobling, W. (2021). Planning for teaching science in the early years. In *Science in early childhood* (pp. 221–238).
- Kaplan, R. M. (1995). Quality of life, resource allocation, and the US health-care crisis. In *Quality of Life and Pharmacoeconomics in Clinical Trials* (2nd ed., pp. 3–30). Lippincott-Raven.

- Badenoch, B., & Bogdan, N. (2012). Safety and connection: The neurobiology of play. In *Play-based interventions for children and adolescents with autism spectrum disorders* (pp. 3–18). Routledge.
- Randall, K. E., Bohnert, A. M., & Travers, L. V. (2021). Understanding affluent adolescent adjustment: The interplay of parental perfectionism, perceived parental pressure, and organized activity involvement. *Journal of Adolescence, 86*, 38–47.
- Shokoohi, R., Hanif, N. R., & Dali, M. (2012). Influence of socio-economic factors on children's school travel. *Procedia – Social and Behavioral Sciences, 50*, 135–147.
- Bradshaw, W. (2013). A framework for providing culturally responsive early intervention services. *Young Exceptional Children, 16*(1), 3–15.
- World Health Organization. (2008). *World report on child injury prevention*. WHO Press.
- World Health Organization. (2019). *WHO guidelines on physical activity, sedentary behaviour and sleep for children under 5 years of age*. WHO Press.
